# Autophagy is dispensable to overcome ER stress in the filamentous fungus *Aspergillus niger*

**DOI:** 10.1002/mbo3.359

**Published:** 2016-03-29

**Authors:** Anne‐Marie Burggraaf, Arthur F. J. Ram

**Affiliations:** ^1^ Molecular Microbiology and BiotechnologyInstitute of Biology LeidenLeiden UniversitySylviusweg 722333 BELeidenThe Netherlands

**Keywords:** *atg1*, endoplasmic reticulum, ERAD, misfolded protein, secretory protein, UPR.

## Abstract

Secretory proteins are subjected to stringent quality control systems in the endoplasmic reticulum (ER) which include the targeting of misfolded proteins for proteasomal destruction via the ER‐associated degradation (ERAD) pathway. Since deletion of ERAD genes in the filamentous fungus *Aspergillus niger* had hardly any effect on growth, this study investigates whether autophagy might function as an alternative process to eliminate misfolded proteins from the ER. We generated *A. niger* double mutants by deleting genes essential for ERAD (*derA*) and autophagy (*atg1* or *atg8*), and assessed their growth both under normal and ER stress conditions. Sensitivity toward ER stress was examined by treatment with dithiothreitol (DTT) and by expressing a mutant form of glucoamylase (mtGlaA::GFP) in which disulfide bond sites in GlaA were mutated. Misfolding of mtGlaA::GFP was confirmed, as mtGlaA::GFP accumulated in the ER. Expression of mtGlaA::GFP in ERAD and autophagy mutants resulted in a twofold higher accumulation in *ΔderA* and *ΔderAΔatg1* strains compared to *Δatg1* and wild type. As *ΔderAΔatg1* mutants did not show increased sensitivity toward DTT, not even when mtGlaA::GFP was expressed, the results indicate that autophagy does not act as an alternative pathway in addition to ERAD for removing misfolded proteins from the ER in *A. niger*.

## Introduction

Folding and post‐translational modification of secretory and transmembrane proteins in eukaryotic cells takes place in the endoplasmic reticulum (ER), with the assistance of chaperones, foldases, and lectins. Stringent quality control mechanisms tightly regulate the folding process, only allowing correctly folded, completely assembled proteins to exit the ER for subsequent delivery to the site of action (Ruggiano et al. 2014). The presence of improperly folded proteins in the ER induces a set of signaling pathways known as the unfolded protein response (UPR) to alleviate the stress and restore homeostasis. Efficient clearance of improperly folded proteins is indispensable for cellular function, since persistent accumulations of unfolded proteins are potentially harmful for the cell as they impair ER function and homeostasis and eventually lead to the activation of apoptotic pathways. The decline in activity of the ER folding machinery with age has been shown to contribute to multiple aging‐related neurodegenerative human diseases, including Alzheimer's, Parkinson's, and Huntington's diseases (Vilchez et al. 2014).

Activation of the UPR upon ER stress results in induced expression of genes encoding chaperones and foldases which enhance protein folding and thereby increase folding capacity. Simultaneously, the UPR regulates the reduction of the ER folding load by the attenuation of protein synthesis on the one hand and the increase of misfolded protein clearance via the ER‐associated degradation (ERAD) system on the other hand (Travers et al. 2000; Ron and Walter 2007). Proteins that are targeted for destruction via ERAD are retrotranslocated to the cytosol for degradation by the 26S proteasome. Studies in yeast and mammalian cells indicate that recognition, retrograde transport, and ubiquitination of misfolded proteins that accumulate in the ER lumen are mediated by the Hrd1 membrane complex, which includes the E3‐ubiquitin ligase Hrd1 itself as well as Hrd3, Der1, and other regulatory and scaffold proteins (Vembar and Brodsky 2008; Ruggiano et al. 2014). Homologs of ERAD genes were also identified in filamentous fungi and studying deletion mutants of a number of those in *Aspergilli* species showed that a functional ERAD pathway is not required for growth both under normal and ER stress conditions (Carvalho et al. 2011a; Krishnan et al. 2013). Deletion of the *der1* homolog *derA* in a heterologous protein overexpression strain of *Aspergillus niger* resulted in a sixfold increase in the intracellular accumulation of the protein accompanied by the induction of UPR target genes, but had no effect on growth and conidiation (Carvalho et al. 2011a).

Clearance of aberrant proteins from the ER can alternatively involve vacuolar targeting via the autophagic pathway (Cheng 2011; Deegan et al. 2013; Pu and Bassham 2013; Senft and Ronai 2015). Autophagy is a highly conserved, catabolic process responsible for the delivery of proteins, cytoplasmic components, and organelles to lytic compartments such as lysosomes in mammalian cells or vacuoles in plant and fungal cells. It involves the sequestering of cytoplasmic contents in double membrane vesicles which subsequently fuse with the lysosome or vacuole, releasing their cargo. Hydrolytic enzymes facilitate the degradation of the vesicle membrane and its contents, whereupon the breakdown products are transported back into the cytoplasm to be reused by the cell (Yang et al. 2006). Autophagic activity is controlled by a set of more than 30 autophagy‐related (Atg) proteins (Feng et al. 2014) and we previously showed that at least two of them (Atg1 and Atg8) are essential for autophagy in *A. niger* (Nitsche et al. 2013). The serine/threonine protein kinase Atg1 forms a part of the regulatory Atg1 kinase complex in yeast which initiates the autophagic process, while Atg8 is a structural component of autophagic vesicle membranes (Cheong et al. 2008; Inoue and Klionsky 2010). Although deletion of either one of these genes results in severe or complete impairment of conidiation in several filamentous fungal species (Pinan‐Lucarré et al. 2005; Kikuma et al. 2006; Richie et al. 2007; Bartoszewska et al. 2011), phenotypic effects were only modest in *A. niger* (Nitsche et al. 2013). Basal levels of autophagy contribute to the maintenance of cellular homeostasis by eliminating old or damaged organelles and long‐lived proteins. Autophagy is strongly induced by conditions of intracellular or extracellular stress, including nutrient starvation, pathogen invasion, and oxidative stress. Increasing evidence indicates that autophagy can also be induced by ER stress, suggesting that it is functioning in the clearance of aggregated and misfolded proteins. Studies in yeast and in mammalian cells show transcriptional induction of genes related to autophagy upon ER stress (Travers et al. 2000; Kouroku et al. 2007) and autophagy‐mediated removal of aggregated mutant proteins from the ER (Kruse et al. 2006; Fujita et al. 2007; Ishida et al. 2009).

In filamentous fungi, the possible link between ER stress and autophagy has been hardly studied. For *Aspergillus oryzae* it was shown that mutant proteins accumulating in the ER are transported to vacuoles upon starvation in an autophagy‐dependent manner (Kimura et al. 2011). In this study, we investigated whether autophagy is involved in the removal of misfolded proteins from the ER in *A. niger* by deleting genes essential for autophagy in an ERAD‐defective background. Growth of the double knockout strains was compared to single deletion mutants of ERAD and autophagy both under normal conditions and after applying ER stress. Intracellular localization of misfolded proteins was visualized by expressing a disulfide bond‐deleted mutant of the secretory protein glucoamylase (GlaA) and compared between wild‐type, single deletion and double knockout strains. The results indicate that deleting autophagy genes in strains defective for ERAD did not have an additional effect on growth and the level of accumulation of misfolded proteins in the ER.

## Experimental Procedures

### Strains, culture conditions, and molecular techniques

*Aspergillus niger* strains used in this study are listed in Table 1. Strains were cultivated in minimal medium (MM) (Bennett and Lasure 1991) or in complete medium containing 0.1% casamino acids and 0.5% yeast extract in addition to MM. Growth media were supplemented with 10 mmol/L uridine when required. Hygromycin‐resistant transformants were isolated from plates supplemented with 200 *μ*g mL^−1^ hygromycin and 500 *μ*g mL^−1^ caffeine and subsequently purified on plates containing 100 *μ*g mL^−1^ hygromycin.

**Table 1:** *Aspergillus niger* strains used in this study

Strain	Genotype	References
N402	*cspA1* derivative of ATCC9029	Bos et al. (1988)
MA78.6	Δ*kusA::amdS* in N402	Carvalho et al. (2010)
MA169.4	*kusA*::DR*‐amdS‐*DR in AB4.1	Carvalho et al. (2010)
AW12.1	Δ*atg1::hyg* in MA78.6	This study
AW13.1	Δ*atg8::hyg* in MA78.6	This study
MA97.2	Δ*kusA*, Δ*derA::amdS*	Carvalho et al. (2011a)
AW14.2	Δ*atg1::hyg* in MA97.2	This study
AW15.1	Δ*atg8::hyg* in MA97.2	This study
MA134.64	Δ*kusA*, multicopy P*gpdA*‐*gla*_514_‐*gus*	Carvalho et al. (2011a)
AW16.1	Δ*atg1::hyg* in MA134.64	This study
AW17.2	Δ*atg8::hyg* in MA134.64	This study
MA136.18	Δ*kusA*, multicopy *PgpdA‐gla*_514_‐*gus*, Δ*derA::amdS*	Carvalho et al. (2011a)
AW18.6	Δ*atg1::hyg* in MA136.18	This study
AW19.9	Δ*atg8::hyg* in MA136.18	This study
AW27.10	5‐FOA‐resistant derivative of MA97.2	This study
AW28.12	5‐FOA‐resistant derivative of AW12.1	This study
AW30.3	5‐FOA‐resistant derivative of AW14.2	This study
AW47.2	P*gpdA‐*wt*glaA::sgfp‐*T*trpC*, pyrG** in MA169.4	This study
AW48.2	P*gpdA‐*mt*glaA::sgfp‐*T*trpC*, pyrG** in MA169.4	This study
AW49.1	P*gpdA‐*wt*glaA::sgfp‐*TtrpC, pyrG** in AW27.10	This study
AW50.1	P*gpdA‐*mt*glaA::sgfp‐*TtrpC, pyrG** in AW27.10	This study
AW51.1	P*gpdA‐*wt*glaA::sgfp‐*TtrpC, pyrG** in AW28.12	This study
AW52.1	P*gpdA‐*mt*glaA::sgfp‐*TtrpC, pyrG** in AW28.12	This study
AW53.2	P*gpdA‐*wt*glaA::sgfp‐*TtrpC, pyrG** in AW30.3	This study
AW54.1	P*gpdA‐*mt*glaA::sgfp‐*TtrpC, pyrG** in AW30.3	This study
MA141.1	P*gpdA‐glaAG2::sgfp‐HDEL‐*TtrpC, pyrG* in MA70.15	Carvalho et al. (2011b)

All PCR and cloning steps were performed according to standard procedures (Sambrook and Russell 2001). Total RNA was extracted using Trizol reagent (Invitrogen, Breda, The Netherlands) according to manufacturer's instructions. Obtainment of uridine‐requiring strains by counter selection on 5‐Fluoroorotic acid (5‐FOA), transformation of *A. niger*, and genomic DNA extraction were conducted as described by Meyer et al. (2010). Northern and Southern blot analyses were performed as described by Sambrook and Russell (2001), using [*α*‐32P]dATP‐labeled probes synthesized with the DecaLabel DNA labeling kit (Thermo Scientific, Breda, The Netherlands).

Plate growth assays were performed on MM solidified by the addition of 1.5% agar and growth at 30°C and 42°C was monitored for 3 days. Sensitivity of the deletion strains toward ER stress was determined by spotting 1 × 10^4^ spores on solid MM supplemented with 1, 5, or 10 mmol/L dithiothreitol (DTT).

### Generation of double knockout strains

Plasmids carrying the *atg1* or *atg8* deletion cassettes were described previously (Nitsche et al. 2013). Linearized constructs were transformed to *A. niger* strains MA78.6 (*ΔkusA*), MA97.2 (*ΔkusA, ΔderA*), MA134.64 (multicopy glucoamylase‐*β*‐glucuronidase [mcGlaGus], *ΔkusA*), and MA136.18 (mcGlaGus, *ΔkusA, ΔderA*) (Carvalho et al. 2010, 2011a). Homologous integration of the constructs was confirmed by Southern blot analysis (Fig. S1).

### Generation of strains constitutively expressing GFP fusion proteins

The pAN52‐1*Not* vector was used to facilitate constitutive expression of *glaA* and mutant *glaA* fusion proteins from the *Aspergillus nidulans gpdA* promoter. An extra *Not*I restriction site was introduced in the *Xba*I site of pAN52‐1Not to enable the isolation of the fusion constructs via *Not*I digestion in a later stage of the cloning procedure. A phosphorylated oligonucleotide adaptor (5′‐CTAGAGCGGCCGCT‐3′) was heated at 95°C for 2 min and slowly cooled down to room temperature. Three volumes of ice‐cold 70% ethanol were added and after centrifugation the pellet was air‐dried and dissolved in Milli‐Q (MQ) water. The adaptor was then directly ligated in *Xba*I‐opened pAN52‐1*Not*‐gfp (M. Arentshorst et al., unpubl. data), giving rise to plasmid pAW56.

The *glaA::sgfp* fragment was PCR amplified from plasmid pAN56‐2sGFP (Gordon et al. 2000), using primers which introduced restriction sites *Nco*I and *Bam*HI (5′‐CATG*CCATGG*GCTTCCGATCTCTACTCGCCCTG and 5′‐CG*GGATCC*TTACTTGTACAGCTCGTCCATG). The mutated form of *glaA::sgfp* (mt*glaA::gfp*) was synthetically produced at GeneArt^®^ Life Technologies (Carlsbad, CA). Both the wild‐type and the mutated fusion constructs were blunt‐end ligated in the pJET1.2 cloning vector, sequenced, and subsequently isolated as two *Nco*I‐*Nhe*I and *Nhe*I‐*Bam*HI fragments in two double digestions to circumvent the extra *Bam*HI site which is present in the fusion construct. The fragments were cloned between the *gpdA* promoter and *trpC* terminator of the *Nco*I–*Bam*HI‐opened pAW56 plasmid in a three‐way ligation.

The constructs, containing the *gpdA* promoter and *trpC* terminator, were subsequently isolated by *Not*I digestion and cloned into pMA334 (Arentshorst et al. 2015). The pMA334 plasmid has been designed such that (reporter) constructs can be inserted between parts of the *pyrG* partial open reading frame and 3′ flanking region, facilitating efficient targeting of the construct to the *pyrG* locus. The constructs were linearized by *Asc*I digestion before transformation to *A. niger* strains MA169.4 (Δ*kusA, pyrG* ^*−*^), AW27.10 (Δ*kusA*Δ*derA, pyrG* ^*−*^), AW28.12 (Δ*kusA*Δ*atg1, pyrG* ^*−*^), and AW30.3 (Δ*kusA*Δ*derA*Δ*atg1, pyrG* ^*−*^). Single integration of the construct on the *pyrG* locus was confirmed by Southern blot analysis (Fig. S3).

### Microscopy, image processing, and statistical analysis

Confocal images were obtained using a confocal laser scanning microscope (Zeiss Imager, Zeiss, Jena, Germany) equipped with a LSM 5 exciter. For fluorescence intensity measurements, five biological replicate experiments were performed for each individual strain and 10 micrographs were taken per experiment using equal microscope settings. Intersections of hyphae were excluded and the fluorescence intensity within the hyphae was measured using the open source image processing program ImageJ (Abramoff et al. 2004). R statistical computing software (R Foundation for statistical computing, Vienna, Austria) was used for statistical analysis. The data were first tested for normality and for homogeneity of variance with Levene's test. Differences in means were analyzed with analysis of variance (ANOVA) and subsequent post hoc Tukey honest significant difference test when significant differences were observed.

## Results

### ERAD/autophagy double knockout mutants do not show increased sensitivity toward ER stress conditions

To prevent persistent accumulation of misfolded proteins in the ER, improperly folded proteins are efficiently removed and degraded by the ERAD system. Surprisingly, compromising the ERAD pathway in the filamentous fungus *A. niger* only had a modest effect on growth (Carvalho et al. 2011a), indicating that other mechanisms might be of importance to help the cells cope with ER stress.

In order to investigate the possible involvement of the autophagy process in the removal of misfolded proteins from the ER, we deleted the autophagy‐essential genes *atg1* and *atg8* in ERAD‐defective *A. niger* Δ*derA* background strains expressing multiple copies of the heterologous GlaGus gene fusion. It has been shown previously that in this strain, to which we will refer to as the mcGlaGus strain, both genes related to ERAD and UPR were transcriptionally induced, which was accompanied by intracellular accumulation of GlaGus (Carvalho et al. 2011a).

The two autophagy genes were deleted in four different background strains: the control strain MA78.6 (Δ*kusA*), the *derA* deletion strain MA97.2 (Δ*kusA*, Δ*derA*), the mcGlaGus strain MA134.64 (Δ*kusA*, mcGlaGus), and the mcGlaGus strain with *derA* deletion MA136.18 (Δ*kusA,* mcGlaGus, Δ*derA*). Transformants for each strain were purified on hygromycin containing medium and homologous integration was confirmed by Southern blot hybridization (Fig. S1).

**Figure 1:**
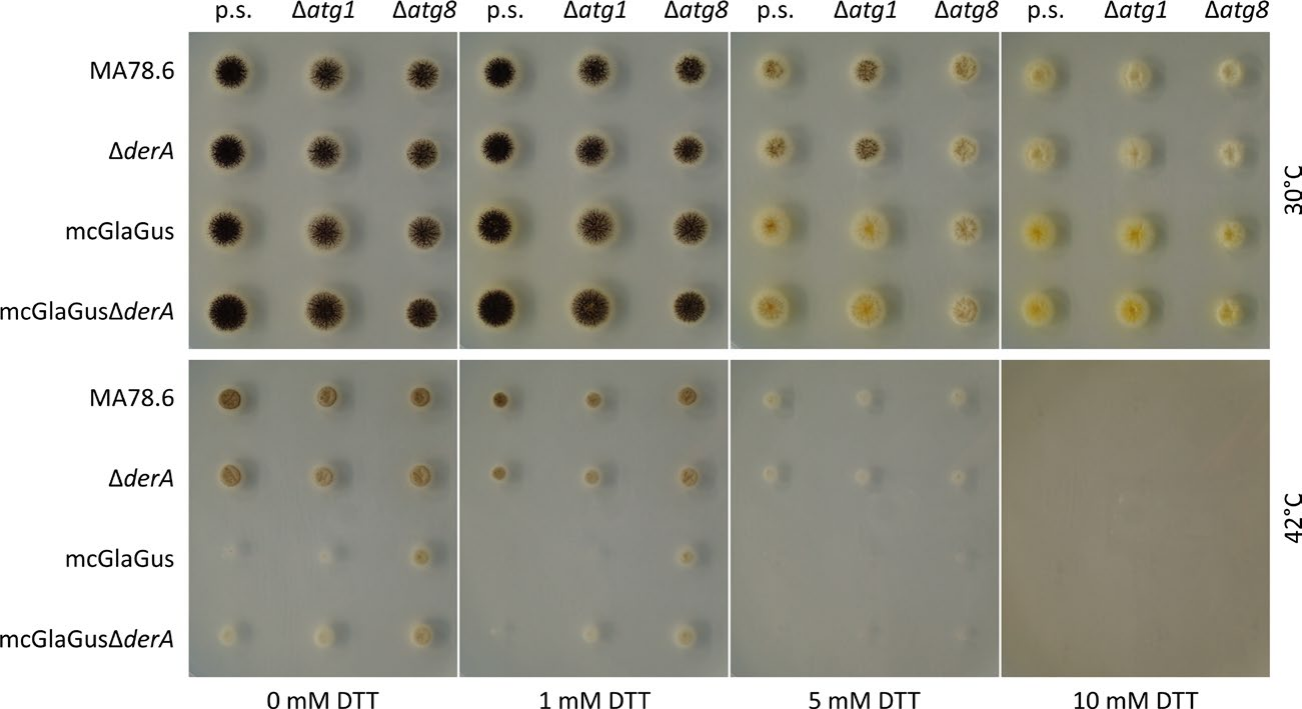
Sensitivity of ERAD and autophagy mutants toward elevated temperature and increasing concentrations of DTT. A total of 10^4^ spores from the parental strains (p.s.) MA78.6, Δ*derA*, mcGlaGus, and mcGlaGusΔ*derA* together with the respective autophagy deletion mutants (Δ*atg1* or Δ*atg8*) was point inoculated on MM agar plates containing 0, 1, 5, or 10 mmol/L DTT and incubated for 3 days at 30°C or 42°C. ERAD, endoplasmic reticulum‐associated degradation; DTT, dithiothreitol; MM, minimal medium.

To study the growth effects of deleting genes related to autophagy and ERAD and compare the double mutants with the single knockout strains, mutants were grown for 3 days on solid MM at 30°C and 42°C (Fig. 1). The mutants were also tested on their sensitivity toward ER stress by exposing them to increasing concentrations of the ER stressor DTT, an inhibitor of disulfide bond formation. Deletion of the *atg* genes in wild‐type and Δ*derA* backgrounds did not result in severe growth defects or increased sensitivity to DTT, although, in accordance with our previous observations, growth and conidiation of the Δ*atg1* and Δ*atg8* mutants was reduced compared to the parental strains (Nitsche et al. 2013). Importantly, no growth reduction was observed in the double knockout mutants compared to the single *atg* deletion strains. Both wild‐type and mutant strains showed sensitivity to elevated temperature and DTT, but no differences were observed among the strains.

Considering the mcGlaGus strains, growth was reduced at 42°C in comparison with the strains that did not produce the heterologous GlaGus fusion protein. It was anticipated that the deletion of both ERAD and autophagy in the background of a heterologous GlaGus protein producing *A. niger* strain would result in serious growth defects. However, no differences considering growth and conidiation between the double mutants and the single mutants could be observed. Even the induction of ER stress through the addition of DTT did not have an effect on the phenotype, as all mutants showed a similar sensitivity to DTT. This indicates that impairment of functional ERAD and autophagy does not affect growth in *A. niger*, not even in heterologous protein overproducing strains.

### The expression of misfolded GlaA::GFP in *A. niger* mildly induces UPR

To study further the role of ERAD and autophagy in the degradation of misfolded proteins in *A. niger*, we investigated whether impairment of these processes causes protein accumulation in the ER by visualizing a wild‐type and a mutant form of the major secreted protein in *A. niger*, GlaA with GFP. The mutant GlaA (mtGlaA) was constructed such that formation of disulfide bridges is prevented in the protein, resulting in improper folding. Crystallographic analysis reveals that the catalytic domain of GlaA contains three disulfide bridges (Lee and Paetzel 2011), which we removed by replacing all the cysteines linked by disulfide bonds with alanines to generate mtGlaA (Fig. S2). Both wild‐type *glaA* (wt*glaA*) and mt*glaA* fused to *sgfp* were constitutively expressed from the *gpdA* promoter and constructs were targeted to the *pyrG* locus in the wild type (MA169.4) and in strains defective for ERAD (Δ*derA*) and/or autophagy (Δ*atg1*, Δ*derA*Δ*atg1*). Single integration at the *pyrG* locus was confirmed by Southern blot analysis (Fig. S3).

**Figure 2:**
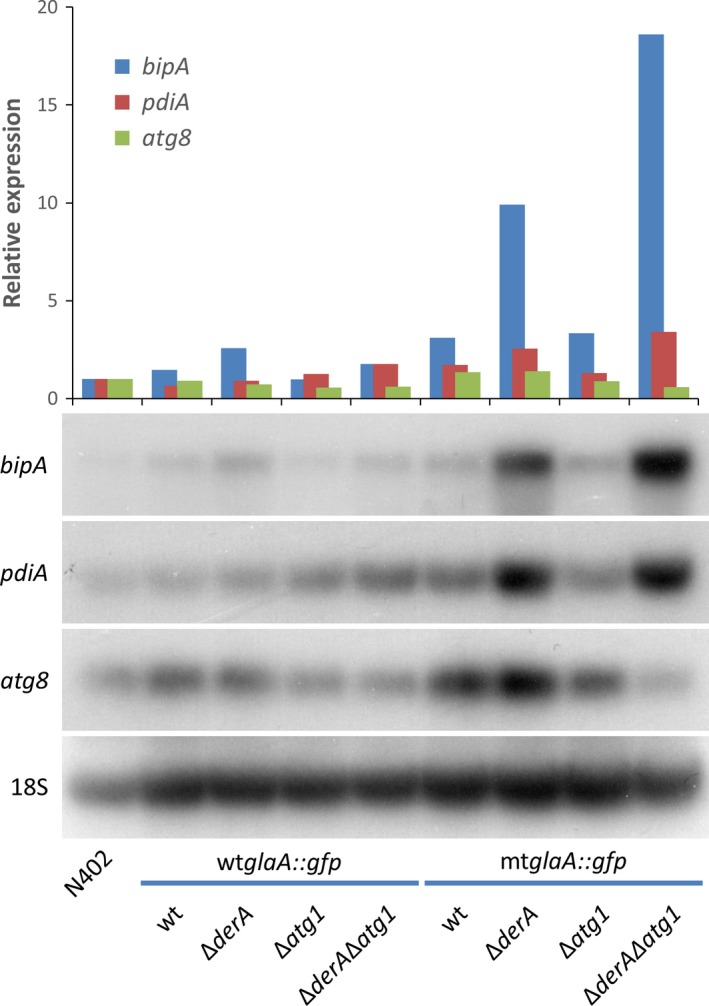
Northern blot analysis of UPR‐ and autophagy‐marker genes in wt*glaA::gfp* and mt*glaA::gfp* expressing strains. Total RNA was extracted after 16 h of growth in liquid CM at 30°C. The gene expression levels were normalized using N402 as a reference. Values were corrected for loading differences by comparison with 18S ribosomal RNA. Strains expressing the misfolded protein mtGlaA::GFP showed an UPR. Wild type (wt), MA169.4. UPR, unfolded protein response; CM, complete medium.

The accumulation of misfolded proteins in the ER induces an ER stress response, characterized by the upregulation of UPR marker genes, such as *bipA* and *pdiA* (Carvalho et al. 2011a). To investigate whether the expression of wtGlaA::GFP or mtGlaA::GFP leads to ER stress, the expression of *bipA* and *pdiA* was determined by Northern blot analysis. In addition, the expression of *atg8* was assessed to see whether autophagy was induced. The results showed that *bipA* and *pdiA* expression levels were comparable between wild type (N402) and strains expressing wt*glaA::gfp*, but were upregulated in mt*glaA::gfp* strains, especially in ERAD‐defective Δ*derA* backgrounds (Fig. 2). This indicates that the presence of misfolded mtGlaA::GFP leads to ER stress, which is further enhanced by the deletion of *derA*. Since *atg8* was not upregulated in strains expressing either wt*glaA::gfp* or mt*glaA::gfp*, it was concluded that autophagy was not activated under these conditions.

### Removal of mtGlaA::GFP from the ER involves ERAD, but is independent of autophagy

**Figure 3:**
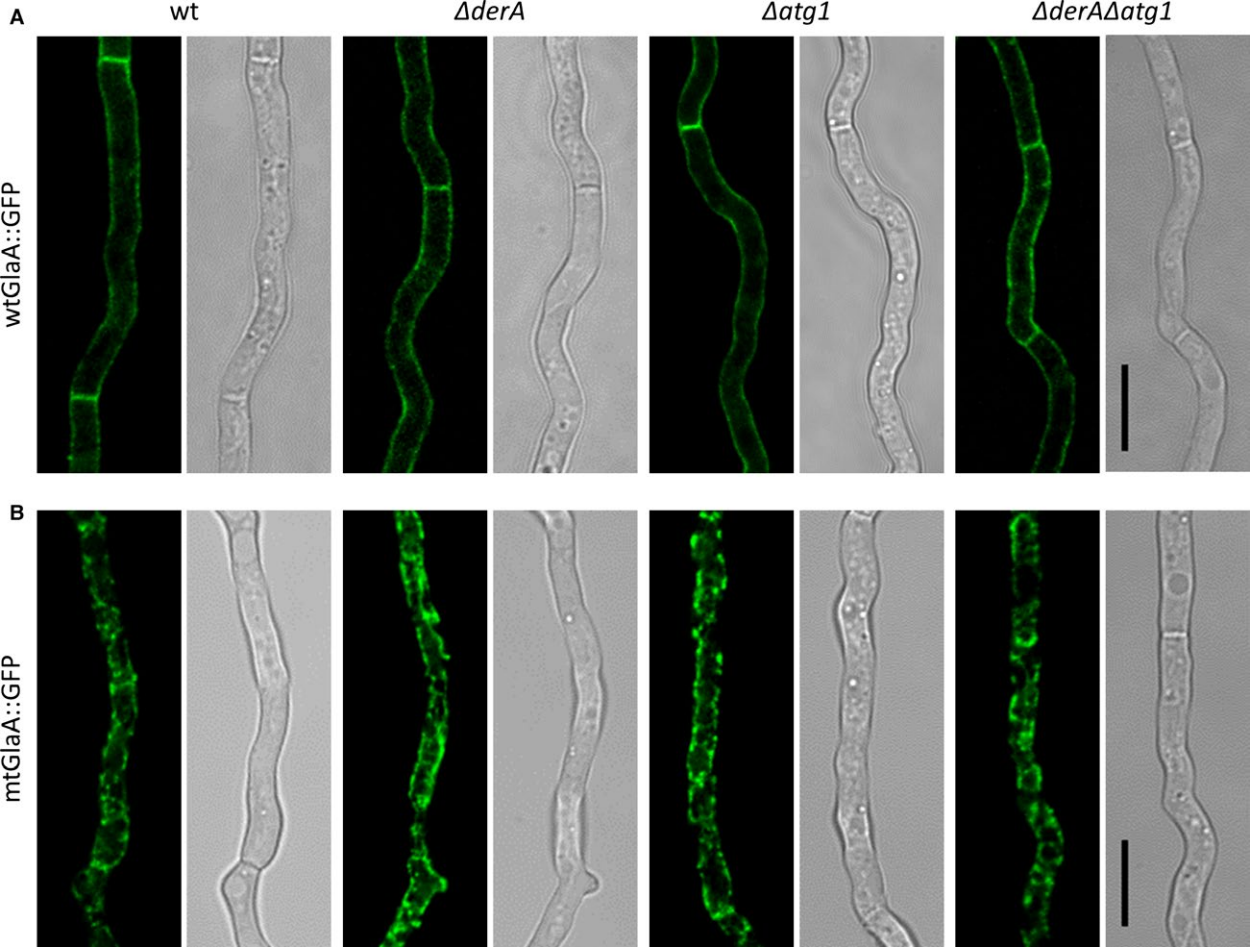
Localization of wtGlaA::GFP and mtGlaA::GFP in wild‐type, Δ*derA*, Δ*atg1*, and Δ*derA*Δ*atg1* background strains. The strains were grown on coverslips in Petri dishes with MES‐buffered MM (pH 6.0) for 16 h at 30°C. Strains expressing wtGlaA::GFP showed localization of GFP in the cell walls and septa (A), whereas GFP localized mainly to the ER in mtGlaA::GFP expressing strains (B). Wild type (wt), MA169.4. Scale bar: 10 *μ*m. MM, minimal medium; ER, endoplasmic reticulum.

Plate growth on MM showed no differences between the strains expressing either wt*glaA::gpf* or mt*glaA::gfp* and their respective parental strains (data not shown).The strains were subsequently subjected to confocal microscopy to investigate the localization of the GFP signal. In the wt*glaA::gfp* strains, fluorescence signal was observed in the cell wall and septa (Fig. 3A), which is typical for secretory proteins and similar as reported before (Gordon et al. 2000). In contrast, mtGlaA::GFP showed an intracellular localization pattern (Fig. 3B), which, by comparison to an *A. niger* strain expressing ER‐targeted GFP (P*glaA*::GFP‐HDEL) (data not shown), was designated as ER localization. This indicates that misfolded GlaA proteins are not transported toward the membrane to be secreted, but at least temporarily retained in the ER to be refolded or degraded.

**Figure 4:**
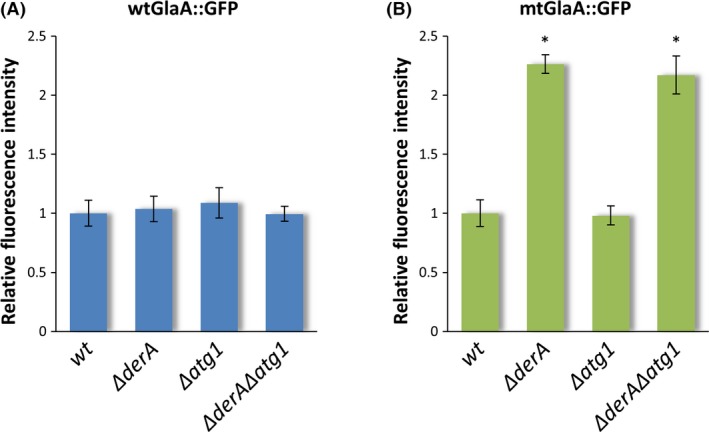
Quantification of wtGlaA::GFP and mtGlaA::GFP fluorescence intensity. The strains were grown on coverslips in Petri dishes with MES‐buffered minimal medium (pH 6.0) for 16 h at 30°C. For each individual strain, the average fluorescence intensity was determined from five independent replicate experiments (*n* = 5) in which 10 images were taken per sample. The intensity levels were normalized to the respective wild‐type values. Strains expressing wtGlaA::GFP showed similar levels of fluorescence (A), while mtGlaA::GFP showed twofold higher accumulation in Δ*derA* and Δ*derA*Δ*atg1* compared to wild‐type (wt, MA169.4) and Δ*atg1* backgrounds (B). Error bars represent standard deviation. *Significantly different from respective wild type (*P* < 0.05).

The degradation of misfolded proteins from the ER is important to prevent harmful accumulations impairing cellular functions. To investigate whether the degradation of misfolded proteins is dependent on functional ERAD and/or autophagy systems, the accumulation of misfolded GlaA proteins in the ER was determined by quantifying the intensity of GFP fluorescence signal from images taken after 16 h of growth. For comparison, strains expressing wtGlaA::GFP proteins were taken along in the analysis and it was observed that all of these strains exhibited the same level of fluorescence (Fig. 4A). However, significant differences were observed among mt*glaA::gfp* strains, with a more than twice as intense signal in the Δ*derA* and the Δ*derA*Δ*atg1* background strains compared to wild type and Δ*atg1* (Fig. 4B). Increased GFP levels in Δ*derA* background strains suggest that lacking a functional ERAD system hampers the efficient degradation of mtGlaA::GFP, leading to accumulation of the misfolded protein in the ER. In contrary, fluorescence intensity of mtGlaA::GFP in the Δ*atg1* background was not different from mtGlaA::GFP in wild type, indicating that degradation of misfolded proteins is independent of functional autophagy. Moreover, even in the absence of ERAD, autophagy is not redundantly taking over its degradation function, since Δ*derA* and Δ*derA*Δ*atg1* displayed similar fluorescence intensities.

### Carbon starvation‐induced transport of wtGlaA::GFP and mtGla::GFP is mediated by autophagy

**Figure 5:**
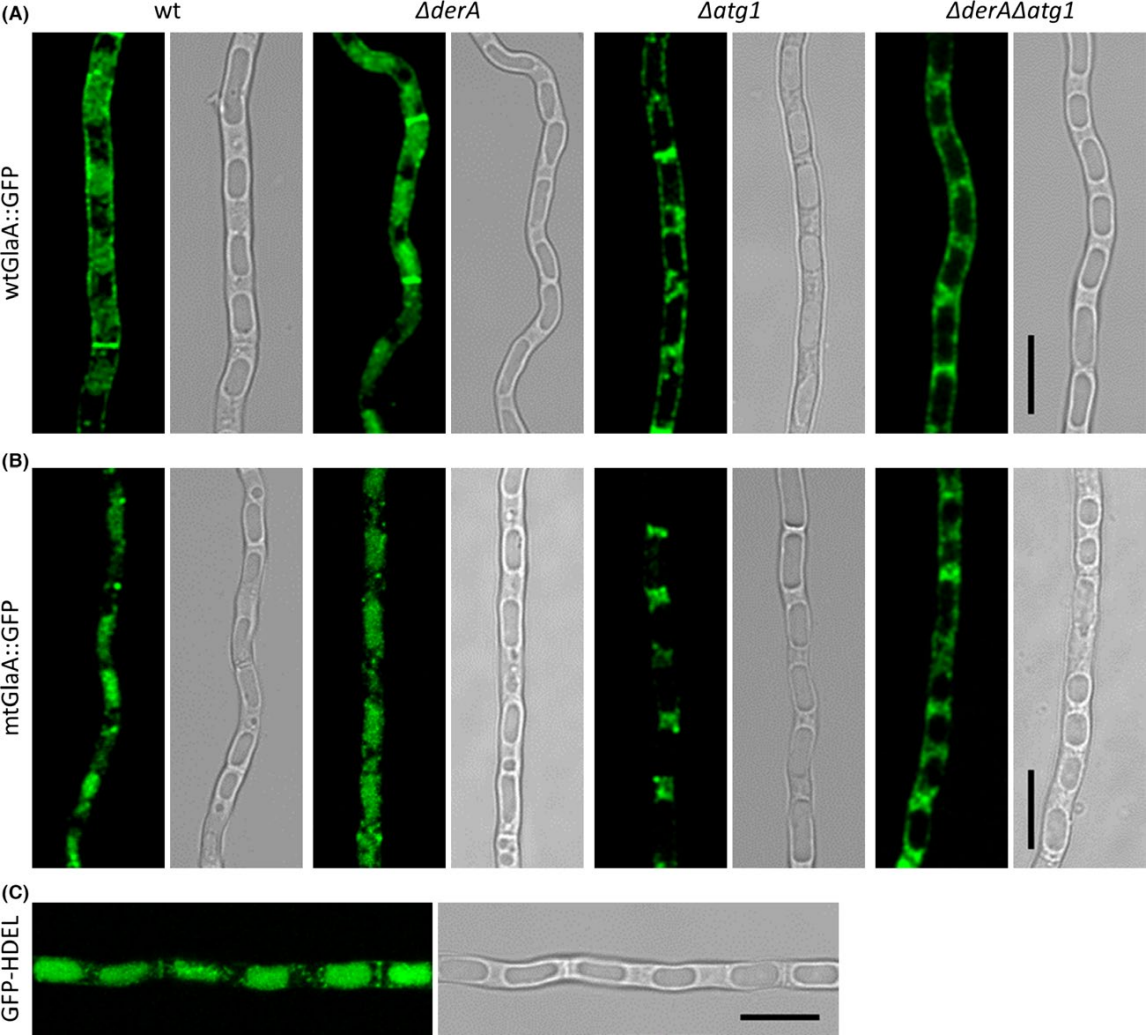
Localization of wtGlaA::GFP, mtGlaA::GFP and GFP‐HDEL during carbon starvation. The strains were pregrown on coverslips in Petri dishes with MES‐buffered minimal medium (MM) (pH 6.0) for 7 h at 30°C. Subsequently, the hyphae were washed and grown in MES‐buffered MM (pH 6.0) without glucose for 40 h at 30°C. Both wtGlaA::GFP (A) and mtGlaA::GFP (B) localized to vacuoles during starvation in wild‐type (wt, MA169.4) and Δ*derA* backgrounds, but not in strains bearing the *atg1* deletion. GFP‐HDEL also localized to the vacuoles (C). Scale bar: 10 *μ*m.

Plate growth assays and fluorescence microscopy described above showed that autophagy is not essential for the degradation of mtGlaA::GFP under normal growth conditions or conditions disturbing protein folding in *A. niger*. We next examined the role of autophagy during carbon starvation. Previously, we demonstrated that both cytosolically and mitochondrially targeted GFP are localized to vacuoles under starvation conditions in *A. niger* wild‐type strains, but not in ∆*atg1* and ∆*atg8* mutants (Nitsche et al. 2013). In order to assess whether wtGlaA::GFP and mtGlaA::GFP are transported to vacuoles upon starvation, the fluorescent strains were subjected to microscopy after 40 h of starvation in MM without a carbon source. Vacuolar localization of both properly folded (wtGlaA::GFP) and misfolded GlaA::GFP proteins was observed in the wild‐type background and in Δ*derA*, but not in the Δ*atg1* and Δ*derA*Δ*atg1* background strains (Fig. 5A and B), indicating that both wtGlaA::GFP and mtGlaA::GFP proteins are delivered to vacuoles upon starvation, which is mediated by autophagy. Subsequent examination of an *A. niger* strain expressing ER‐targeted GFP under the same conditions showed localization of GFP in the vacuole (Fig. 5C), suggesting that parts of the ER are delivered to vacuoles for degradation and recycling upon carbon starvation. Taken together, these results indicate that the transport of both wtGlaA::GFP and mtGlaA::GFP to the vacuole in response to carbon starvation is mediated via the autophagic pathway and is likely to occur as a consequence of bulk degradation of ER, and not specific for misfolded proteins in the ER.

## Discussion

In this study, we investigated the effects of defective ERAD and autophagy on the degradation of misfolded secretory proteins in *A. niger*. The natural property of filamentous fungi to produce and secrete large amounts of proteins makes them attractive hosts for the industrial production of extracellular proteins. Approaches to further improve production yields have been successfully taken for the production of homologous proteins, but the secretion capacity for heterologous proteins is to date far lower (Nevalainen and Peterson 2014). Besides the extracellular activity of efficiently produced secreted proteases (van den Hombergh et al. 1997; Punt et al. 2008; Braaksma et al. 2009; Yoon et al. 2009, 2011), low product yields can also be the result of intracellular degradation of the protein, as a consequence of the strict quality control in the ER which eventually eliminates proteins that fail proper folding or processing (Jacobs et al. 2009). Increased heterologous protein production has been reported upon the overexpression of genes encoding cellular foldases and chaperones in several *Aspergillus* species (reviewed by Heimel 2015) and upon the deletion of some autophagy genes in *A. oryzae* (Yoon et al. 2013). Deletion of ERAD components *derA* and *hrdC* in *A. niger* resulted in an increase in the intracellular accumulation of the heterologous Gla::Gus fusion protein, but had no severe effect on the growth phenotype (Carvalho et al. 2011a). Since persistent accumulations of misfolded proteins are considered harmful to cells by causing severe ER stress, the modest phenotypic effect of compromising ERAD components in *A. niger* was surprising and suggests the presence of an alternative degradation mechanism.

A number of studies have pointed at the importance of autophagy in the degradation of misfolded proteins accumulating in the ER. Several disease‐associated mutant proteins have been shown to be removed from the ER via autophagy. Accumulation of a mutant form of the human dysferlin transmembrane protein in the ER was increased after treatment with lysosome inhibitors and in atg5‐deficient mice cells, whereas accumulation was inhibited upon induction of the autophagy pathway by rapamycin (Fujita et al. 2007). Likewise, degradation of excess *α*1‐antitrypsin Z aggregates, which have been associated with the development of chronic liver disease, is dependent on a functional autophagy machinery in yeast and mice cells (Kamimoto et al. 2006; Kruse et al. 2006). Furthermore, cytosolic aggregates of expanded polyglutamine (polyQ) are degraded via ER stress‐mediated autophagy (Kouroku et al. 2007). Surprisingly, the misfolded protein constructed in this study (mtGlaA::GFP) showed increased ER‐accumulation in the ERAD‐defective strain (Δ*derA*), but not in the autophagy mutant (Δ*atg1*) (Fig. 4). The double knockout mutant exhibited accumulation levels similar to Δ*derA*, indicating that ERAD is involved in the degradation of misfolded proteins but autophagy is not. Even in the absence of functional ERAD, autophagy is not alternatively taking over its functions in degrading misfolded proteins from the ER.

Gene expression levels of UPR target genes *bipA* and *pdiA* were higher in strains expressing the misfolded mtGlaA::GFP protein compared to the N402 strain (Fig. 2). This suggests the presence of ER stress and has also been demonstrated upon overexpression of the heterologous GlaA::Gus protein in *A. niger* (Carvalho et al. 2011a) and upon expression of a mutant form of cellobiohydrolase I in *Trichoderma reesei* (Kautto et al. 2013). Strains bearing a *derA* deletion showed higher induction than wild‐type and Δ*atg1* single knockout strains, indicating a stronger UPR in ERAD‐defective backgrounds. However, the observed UPR seemed rather mild and therefore it cannot be excluded that the ER stress caused by the accumulation of mtGlaA::GFP is not sufficient to induce the autophagy pathway. Such an effect has been observed for the *α*1‐antitrypsin Z protein, as degradation was dependent on autophagy only when this ERAD substrate was overexpressed (Kruse et al. 2006). Interestingly, mutant procollagen trimers are degraded via autophagy, while misfolded procollagen monomers are eliminated through the ERAD pathway (Ishida et al. 2009), suggesting that autophagy is induced as an ultimate strategy for cell survival to remove protein aggregates which cannot be degraded by the ERAD system. Further research is required to determine whether further elevated expression levels (e.g., by constructing multicopy strains) of mtGlaA::GFP are able to induce the process of autophagy in *A. niger*. However, previous experiments have shown that the presence of severe ER stress upon constitutive expression of *hacA* or after treatment with either DTT or tunicamycin did not result in transcriptional induction of the autophagy process in *A. niger* (Guillemette et al. 2007; Carvalho et al. 2012). In addition, our observation that the Δ*derA*Δ*atg1* double mutant expressing mtGlaA::GFP is equally sensitive to the ER stress inducing compound DTT, further supports the conclusion that autophagy is dispensable to overcome ER stress in *A. niger*.

Induction of the autophagy process upon starvation has been demonstrated in many organisms, including *A. niger* (Nitsche et al. 2012). To elucidate whether misfolded proteins are targeted to vacuoles under autophagy‐inducing conditions, wtGlaA::GFP and mtGlaA::GFP strains were microscopically examined after 40 h of carbon starvation. The results show that both wtGlaA::GFP and mtGlaA::GFP proteins are transported to vacuoles in an autophagy‐dependent manner (Fig. 5A and B). Kimura et al. (2011) demonstrated that a misfolded form of the *α*‐amylase protein was delivered to vacuoles via autophagy in late phase cultures of *A. oryzae*. However, in contrast to the observations reported in that study, we found wtGlaA::GFP also being targeted to the vacuoles dependent on autophagy, indicating that vacuolar targeting of proteins from the ER is not specific to misfolded proteins only. During starvation conditions, recycling of cytosolic contents and organelles via autophagy is important for the cell to survive. In this context, the ER can become a substrate of autophagic degradation, which is a specific type of autophagy known as ER‐phagy or reticulophagy (Kraft et al. 2009). Recently, Atg39 and Atg40 were identified as receptor proteins mediating this process in yeast and mammalian cells (Khaminets et al. 2015; Mochida et al. 2015). No homologs of Atg39 or Atg40 were identified in the *A. niger* genome by BLASTp analysis, but in this study we provide evidence for the presence of ER‐phagy (Fig. 5C). Taken together, these data suggest that the autophagy‐dependent delivery of proteins from the ER to the vacuoles upon starvation is likely the result of bulk degradation of ER and its contents.

In this study, a fluorescently labeled misfolded secretory protein was successfully expressed as a reporter in *A. niger*, which is a powerful tool to study the secretory pathway and the response to accumulations of improperly folded proteins in the ER. By expressing this mutant protein in different *A. niger* mutants, we were able to show that autophagy is not specifically involved in the degradation of misfolded proteins from the ER. Surprisingly, the ERAD/autophagy double knockout mutant expressing the misfolded protein was not impaired in growth, although it accumulated the mutant protein in the ER. Since intracellular protein accumulations are normally highly detrimental for cellular functions, it seems likely that the degradation of misfolded mtGlaA::GFP proteins is not completely impaired in Δ*derA* and Δ*derA*Δa*tg1* strains. Other mechanisms might be involved to limit protein accumulations to levels which *A. niger* is able to cope with. In this study, we showed that autophagy does not play a role in this process, and we consider vacuolar targeting independent of the autophagic pathway as unlikely, as we did not observe transport of mtGlaA::GFP to vacuoles in *atg1* deletion strains. Yet another possibility is proteasomal degradation independent of DerA. In *A. fumigatus* it has been demonstrated that deletion of the ERAD gene *hrdA* in addition to the deletion of *derA* highly increased the susceptibility to ER stressing agents such as tunicamycin (Krishnan et al. 2013), indicating that DerA and HrdA have synergistic functions. Further studies are required to explore the proteasomal pathway either by making double knockout mutants of the ERAD system or by applying proteasome inhibitors like MG132 (Kautto et al. 2013) and the fluorescent mtGlaA::GFP protein could be used as a reporter to monitor protein accumulations under these conditions.

## Conflict of Interest

None declared.

## Supporting information


**Figure S1.** Southern blot analysis for *atg* deletions in MA78.6, MA97.2, MA134.64, and MA136.18 backgrounds using hygromycin as a selection marker. The restriction sites used are indicated in the schematic drawings below the photographs. The dashed lines indicate the probes. (A) Genomic DNA of putative Δ*atg1* transformants was digested with *EcoR*I. Expected band sizes for wild type (wt) and mutant are 2.1 and 3.0 kb, respectively. The asterisks indicate the selected transformants, which were named AW12.1, AW14.2, AW16.1, and AW18.6, respectively. (B) Genomic DNA of putative Δ*atg8* transformants was digested with *Sal*I. Expected band sizes for wild type and mutant are 2.5 and 4.2 kb, respectively. The selected transformants are indicated with an asterisks and were named AW13.1, AW15.1, AW17.2, and AW19.9, respectively.
**Figure S2.** Amino acid sequence of the *Aspergillus niger* glucoamylase protein. Three disulfide bridges are present in the catalytic domain. Cysteine residues linked by disulfide bonds are indicated with asterisks.
**Figure S3.** Southern blot analysis for the integration of P*gpdA*‐wt*glaA::gfp*‐T*trpC*‐pyrG** and P*gpdA*‐mt*glaA::gfp*‐T*trpC*‐pyrG** constructs on the pyrG locus in parental strains (p.s.) MA169.4, AW27.10, AW28.12, and AW30.3. Restriction sites of the used enzyme *Xba*I are indicated in the schematic drawing below the photographs. The probe is indicated with a dashed line and the dots indicate mutated cysteine residues involved in disulfide bond formation. For parental strains, a 3.9‐kb band is expected, whereas for mutant strains a 8.2‐kb band is expected. Selected mutant strains are indicated with an asterisk. Strains containing the wt*glaA::gfp* constructs were named AW47.2, AW49.1, AW51.1, and AW53.2. Strains containing the mt*glaA::gfp* construct were named AW48.2, AW50.1, AW52.1, and AW54.1.
