# Supplementary material for: Autophagy is dispensable to overcome ER stress in the filamentous fungus Aspergillus niger
Source: Microbiologyopen. 2016 Mar 29;5(4):647–58. doi: 10.1002/mbo3.359 (PMC4985598; doi:10.1002/mbo3.359)
Supplement: Supplementary file 1 — Figure S1. Southern blot analysis for atg deletions in MA78.6, MA97.2, MA134.64, and MA136.18 backgrounds using hygromycin as a selection marker. The restriction sites used are indicated in the schematic drawings below the photographs. The dashed lines indicate the probes. (A) Genomic DNA of putative Δatg1 transformants was digested with EcoRI. Expected band sizes for wild type (wt) and mutant are 2.1 and 3.0 kb, respectively. The asterisks indicate the selected transformants, which were named AW12.1, AW14.2, AW16.1, and AW18.6, respectively. (B) Genomic DNA of putative Δatg8 transformants was digested with SalI. Expected band sizes for wild type and mutant are 2.5 and 4.2 kb, respectively. The selected transformants are indicated with an asterisks and were named AW13.1, AW15.1, AW17.2, and AW19.9, respectively. Figure S2. Amino acid sequence of the Aspergillus niger glucoamylase protein. Three disulfide bridges are present in the catalytic domain. Cysteine residues linked by disulfide bonds are indicated with asterisks. Figure S3. Southern blot analysis for the integration of PgpdA‐wtglaA::gfp‐TtrpC‐pyrG** and PgpdA‐mtglaA::gfp‐TtrpC‐pyrG** constructs on the pyrG locus in parental strains (p.s.) MA169.4, AW27.10, AW28.12, and AW30.3. Restriction sites of the used enzyme XbaI are indicated in the schematic drawing below the photographs. The probe is indicated with a dashed line and the dots indicate mutated cysteine residues involved in disulfide bond formation. For parental strains, a 3.9‐kb band is expected, whereas for mutant strains a 8.2‐kb band is expected. Selected mutant strains are indicated with an asterisk. Strains containing the wtglaA::gfp constructs were named AW47.2, AW49.1, AW51.1, and AW53.2. Strains containing the mtglaA::gfp construct were named AW48.2, AW50.1, AW52.1, and AW54.1. [file MBO3-5-647-s001.pdf]

Supplementary material

Autophagy is dispensable to overcome ER stress in the filamentous fungus *Aspergillus niger*

Anne-Marie Burggraaf[1] and Arthur F.J. Ram[1*]

[1]Leiden University, Institute of Biology Leiden, Molecular Microbiology and Biotechnology, Sylviusweg 72, 2333 BE Leiden, The Netherlands

[*]Corresponding author: a.f.j.ram@biology.leidenuniv.nl

Tel. (+31) 71 5274914; Fax (+31) 71 5274999

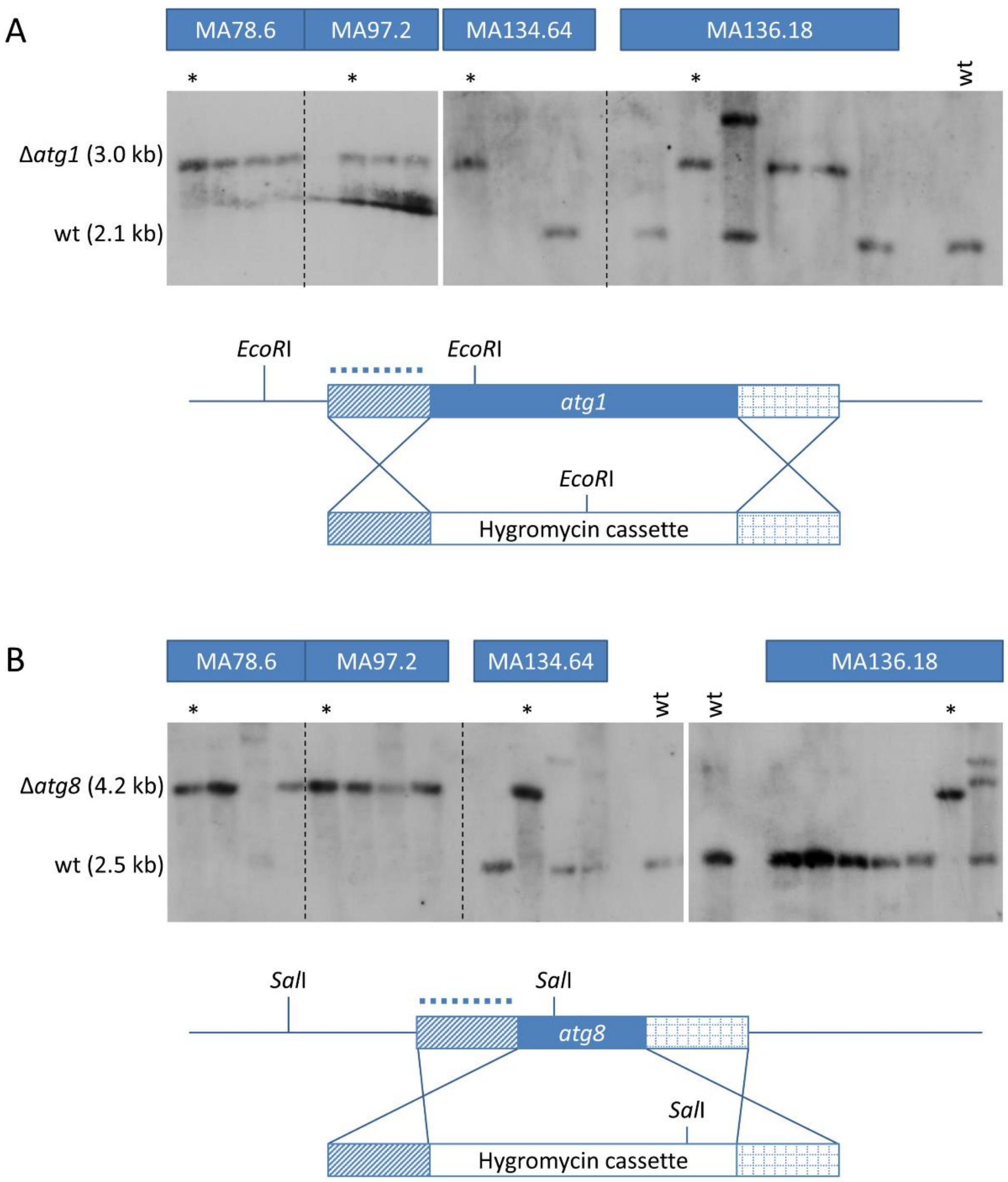

Fig. S1 Southern analysis for *atg* deletions in MA78.6, MA97.2, MA134.64 and MA136.18 backgrounds using hygromycin as a selection marker. The restriction sites used are indicated in the schematic drawings below the photographs. The dashed lines indicate the probes. **A)** Genomic DNA of putative Δ*atg1* transformants was digested with *EcoR*I. Expected band sizes for wild-type (wt) and mutant are 2.1 kb and 3.0 kb, respectively. The asterisks indicate the selected transformants, which were named AW12.1, AW14.2, AW16.1 and AW18.6 respectively. **B)** Genomic DNA of putative Δ*atg8* transformants was digested with *Sal*I. Expected band sizes for wild-type and mutant are 2.5 kb and 4.2 kb respectively. The selected transformants are indicated with an asterisks and were named AW13.1, AW15.1, AW17.2 and AW19.9 respectively.

1 MSFRSLLALS GLVCTGLANV ISKRATLDSW LSNEATVART AILNNIGADG AWVSGADSGI VVASPSTDNP DYFYTWTRDS

81 GLVLKTLVDL FRNGDTSLLS TIENYISAQA IVQGISNPSG DLSSGAGLGE PKFNVDETAY TGSWGRPQRD GPALRATAMI

161 GFGQWLLDNG YTSTATDIVW PLVRNDLSYV AQYWNQTGYD LWEEVNGSSF FTIAVQHRAL VEGSAFATAV GSSCSWCDSQ

241 APEILCYLQS FWTGSFILAN FDSSRSGKDA NTLLGSIHTF DPEAACDDST FQPCSPRALA NHKEVVDSFR SIYTLNDGLS

321 DSEAVAVGRY PEDTYYNGNP WFLCTLAAAE QLYDALYQWD KQGSLEVTDV SLDFFKALYS DAATGTYSSS SSTYSSIVDA

401 VKTFADGFVS IVETHAASNG SMSEQYDKSD GEQLSARDLT WSYAALLTAN NRRNSVVPAS WGETSASSVP GTCAATSAIG

481 TYSSVTVTSW PSIVATGGTT TTATPTGSGS VTSTSKTTAT ASKTSTSTSS TSCTTPTAVA VTFDLTATTT YGENIYLVGS

561 ISQLGDWETS DGIALSADKY TSSDPLWYVT VTLPAGESFE YKFIRIESDD SVEWESDPNR EYTVPQACGT STATVTDTWR

Fig. S2 Amino acid sequence of the *A. niger* glucoamylase protein. Three disulfide bridges are present in the catalytic domain. Cysteine residues linked by disulfide bonds are indicated with asterisks.

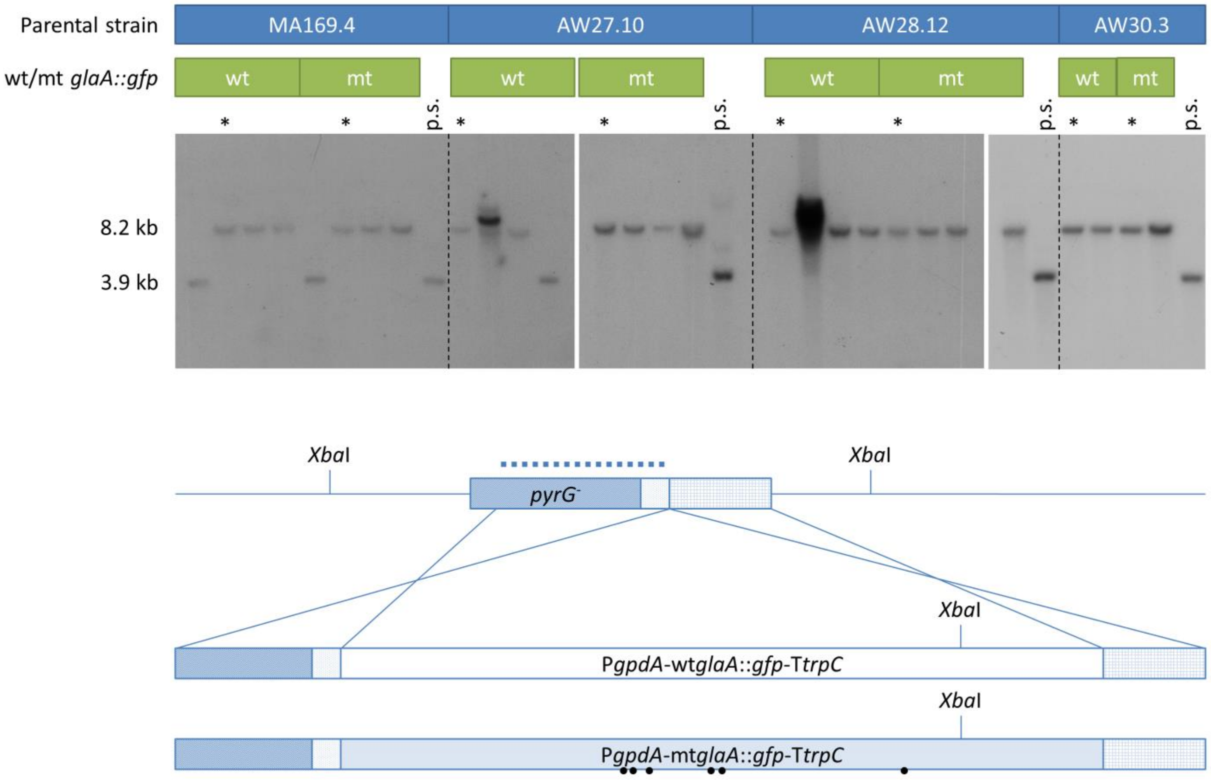

Fig. S3 Southern analysis for the integration of P*gpdA*-wt*glaA::gfp*-T*trpC*-*pyrG*** and P*gpdA*-mt*glaA::gfp*-T*trpC*-*pyrG*** constructs on the pyrG locus in parental strains (p.s.) MA169.4, AW27.10, AW28.12 and AW30.3. Restriction sites of the used enzyme *Xba*I are indicated in the schematic drawing below the photographs. The probe is indicated with a dashed line and the dots indicate mutated cysteine residues involved in disulphide bond formation. For parental strains a 3.9 kb band is expected, whereas for mutant strains a 8.2 kb band is expected. Selected mutant strains are indicated with an asterisk. Strains containing the wt*glaA::gfp* constructs were named AW47.2, AW49.1, AW51.1 and AW53.2. Strains containing the mt*glaA::gfp* construct were named AW48.2, AW50.1, AW52.1 and AW54.1.
